# College and Hospital Notes

**Published:** 1902-12

**Authors:** 


### COLLEGE AND HOSPITAL NOTES.

---

Three new buildings are being erected near the Michigan University Hospital, Ann Arbor. One, the psychopathic ward, is to cost $50,000. The appropriation was obtained from the state legislature largely through the efforts of Dr. William J. Herdman. The first stages of insanity will be studied here. The state has generously provided for the maintenance of the ward.

The late Mrs. L. M. Palmer bequeathed $20,000 for the establishment of a ward for children. The building which is to be known as the "Palmer ward" is fast approaching completion. Mrs. Palmer also bequeathed $15,000 as an endowment for the maintenance of this ward.

A large new boiler house is also being erected. The old boiler has been removed from the basement of the nurses' home.

---

During the last school year 3,624 volumes were presented as gifts to the libraries of the University of Michigan. The total additions to the several libraries for the year were 9,539 volumes. Over seven thousand of these went into the general library, over one thousand into the medical library, and eight hundred into the law library.

---

The Daughters of the Confederacy have decided to purchase the Stonewall Jackson homestead, at Lexington, Va., and convert it into the Jackson Memorial Hospital.

